# A case of tumor lysis syndrome and acute renal failure associated with elotuzumab treatment in multiple myeloma

**DOI:** 10.5414/CNCS109165

**Published:** 2017-11-22

**Authors:** Douglas K. Atchison, H. David Humes

**Affiliations:** University of Michigan Health System, Department of Internal Medicine, Division of Nephrology, A. Alfred Taubman Health Care Center, Ann Arbor, MI, USA

**Keywords:** elotuzumab, tumor lysis syndrome, dabigatran, acute kidney injury, multiple myeloma

## Abstract

Renal dysfunction is a common comorbidity of multiple myeloma. However, tumor lysis syndrome is a rare cause of renal dysfunction in multiple myeloma. Elotuzumab is a newly US FDA-approved monoclonal antibody used in the treatment of refractory multiple myeloma. To our knowledge, elotuzumab has not been associated with a case of tumor lysis syndrome. We present the case of a patient who developed clinical tumor lysis syndrome 1 week after treatment with elotuzumab accompanied by renal failure with hyperphosphatemia, hyperkalemia, and profound hyperuricemia. His course was further complicated by significant epistaxis from the accumulation of dabigatran in acute renal failure. In spite of treatment with rasburicase and hemodiafiltration, the patient decompensated and eventually died. Risk factors for the development of tumor lysis syndrome in multiple myeloma are discussed.

## Introduction 

Multiple myeloma (MM) is a clonal B-cell neoplasm that accounts for 10% of hematological malignancies [1]. Renal dysfunction is relatively common in MM, with the more common etiologies being cast nephropathy, light-chain deposition disease, acute tubular necrosis, or even glomerulonephritis. Tumor lysis is a relatively rare cause of renal dysfunction, estimated to occur in ~ 1% of cases of MM in specific case series [2]. 

Tumor lysis syndrome (TLS) is a syndrome characterized by metabolic and electrolyte abnormalities that occur during the rapid breakdown of hyperproliferative malignant cells. Clinical TLS, as classified by the Cairo-Bishop definition, is the occurrence of relative or absolute hyperkalemia, hyperuricemia, hyperphosphatemia, and/or hypocalcemia [3]. Two or more laboratory abnormalities are needed within 3 days prior to, or up to 7 days after starting chemotherapy. Additionally, for clinical TLS to be diagnosed, an elevated serum creatinine (> 1.5 times the upper limit of normal), sudden death, and/or cardiac arrhythmia, or seizure, which is not directly attributable to the chemotherapeutic agent started, must occur. 

As described above, TLS is a relatively rare occurrence in MM. Many of these cases usually occur in a setting of high tumor proliferation [1, 2, 4] or in cases treated with bortezomib [5], melphalan [2], cyclophosphamide [2], or thalidomide [6]. Elotuzumab is a monoclonal antibody against signaling lymphocytic activation factor molecule F7 (SLAMF7), which causes the activation of natural killer cells against the MM cells and direct antibody-mediated cytotoxicity [7]. To our knowledge, there have been no published reports of TLS caused by elotuzumab. 

Here, we report a case of a patient who developed acute renal failure from TLS after receiving elotuzumab in MM. 

## Case report 

The patient was a 61-year-old male who had been diagnosed with IgG-κ MM in 2011, with plasmacytes comprising 45% of the bone marrow. His comorbidities included atrial fibrillation on dabigatran, heart failure with preserved ejection fraction, mitral valve replacement with bioprosthetic valve, right lower extremity deep venous thrombosis (DVT), and benign prostatic hypertrophy. His previous treatment course had consisted of lenalidomide, carfilzomib, and dexamethasone, following autologous stem-cell transplant in 2011, complicated by recurrence of his MM. Additional treatments since then included pomalidomide, cyclophosphamide, and carfilzomib. Most recently, he had been treated with daratumumab from April to October of 2016. Lenalidomide was also added in October. However, both daratumumab and lenalidomide were stopped due to a combination of disease progression (M-protein increasing from 0.6 to 2.2 g/dL) and respiratory infection requiring hospitalization. The patient reinitiated nightly 15 mg of lenalidomide on November 8, 2016 and received his first dose of 800 mg of elotuzumab with 20 mg of dexamethasone on November 15, 2016. 

Other medications at the time of elotuzumab infusion included: acetaminophen 500 – 1,000 mg p.r.n., acyclovir 400 mg b.i.d., benzonatate 200 mg t.i.d. p.r.n., carvedilol 6.25 mg b.i.d., dabigatran etexilate 150 mg b.i.d., diphenhydramine 25 mg b.i.d., eszopiclone 3 mg QHS, finasteride 5 mg daily, levocarnitine 250 mg t.i.d., loperamide 2 mg q.i.d. p.r.n., magnesium carbonate 400 mg b.i.d., ondansetron 8 mg t.i.d. p.r.n., prochlorperazine 10 mg q.i.d. p.r.n., thyroid pork 65 mg daily, zolendronic acid 4 mg Q3 months (last dose October 11, 2017), albuterol 90 µg p.r.n., biotin 500 mg daily, calcium carbonate and vitamin D3 1,500 – 400 units b.i.d., codeine-guaifenesin 20 – 200 mg q.i.d. p.r.n., ferrous sulfate 325 mg daily, hydrocodone-acetaminophen 5 – 325 mg p.r.n., melatonin 3 mg QHS, multivitamins and vitamin B12 (doses unknown), omeprazole 20 mg b.i.d., pentoxifylline 400 mg b.i.d., polyethylene glycol 17 g daily p.r.n., and naproxen 250 mg b.i.d. p.r.n. (self-reported). 

The patient complained of worsening fatigue, decreased oral intake, and exertional shortness of breath in the week following the infusion. He complained of worsening arthralgias, for which he self-initiated treatment with naproxen 250 mg b.i.d.. He presented for his second infusion of elotuzumab on November 22, 2016, but given significantly deranged preinfusion labs, he was directed to the emergency department. There, his initial vitals were: temperature of 36.5 °C, blood pressure 85/48 mmHg, heart rate of 70 beats per minute, 18 respirations per minute, and saturation 94% on room air. His physical exam was remarkable for dry mucous membranes and positive straight leg raise test. 

His serum labs pre- and post-elotuzumab are listed in Table 1. Of note, the patient’s creatinine had increased significantly post-elotuzumab, with newly noted hyperkalemia, hyperphosphatemia, and marked hyperuricemia. His dabigatran level was elevated above the level of detection. His urinary labs were notable for a urinalysis with specific gravity of 1.01, pH of 5.5, negative leukocyte esterase or nitrites, positive for trace protein, trace blood. His urine microscopy was significant for numerous uric acid crystals and hyaline casts. His urine had 2.64 g protein/g creatinine, which was predominantly α- and β-globulins with IgG-κ M-protein detected per urine electrophoresis. His renal ultrasound demonstrated bilateral renal calculi, the largest of which was 5 mm. There was no evidence of obstruction. 

The patient was given an IV fluid challenge, without significant improvement in his plasma creatinine. He received 6 mg of rasburicase while in the emergency department. He remained oliguric and only made 500 cc of urine in his first 24 hours in the hospital. Because of his marked hyperuricemia, hyperkalemia, and oliguria, the patient was initiated on continuous venovenous hemodiafiltration (CVVHDF). Over the next 30 hours, the patient’s uric acid and potassium improved to 1.7 mg/dL and 4.7 mmol/L, respectively. However, the patient’s course was complicated by recurrent large-volume epistaxis that was refractory to dabigatran reversal with idaricizumab, desmopressin, plasma, platelet, and blood transfusions and repeated packing and cautery. He became progressively more lethargic, and his family decided to cease aggressive measures and transferred the patient to home hospice where he expired. 

## Discussion 

To summarize, we presented the case of a 61-year-old male with MM who presented with profound hyperuricemia and uricosuria, hyperkalemia, hyperphosphatemia, and acute renal failure within 1 week of starting elotuzumab therapy for MM. To our knowledge, this represents the first published case of TLS associated with elotuzumab usage in MM. 

Elotuzumab is a monoclonal antibody against SLAMF7, recently approved by the US FDA for treatment of MM. In the recent phase-3 clinical trial, ELOQUENT-2, no mention of TLS in patients treated with elotuzumab is noted in either the adverse events section or supplemental data [7]. Additionally, no mention of TLS or hyperuricemia in a phase-1b study with elotuzumab was reported [8]. 

Tumor lysis syndrome is a relatively rare complication of MM. Our patient met 3 of the 4 criteria (hyperuricemia, hyperkalemia, hyperphosphatemia) of the Cairo-Bishop TLS scoring system, starting 7 days after elotuzumab administration. Moreover, the patient had an elevation of creatinine > 1.5 times the upper limit of normal, thereby meeting the criteria for clinical TLS. Per retrospective single-center case series, the prevalence of TLS in MM was ~ 1 – 10% [2, 4]. All patients in the first case series had bulky bone marrow disease, with none having less than 70% plasma cells on their bone marrow biopsy differential. Additionally, the majority of patients also had either highly proliferative disease (as measured by plasmacyte labeling index) and/or unfavorable cytogenetics. Similar to this, a second retrospective study demonstrated that the presence of circulating plasma cells, higher International Staging System score for MM, higher baseline uric acid and creatinine were all risk factors for the development of TLS [4]. Consistent with this, the recent appearance of circulating plasmacytes on our patient’s blood differential suggests a more highly proliferative state, which may have been his major risk factor for TLS development. Rare cases of TLS have been reported with most of the other chemotherapeutic treatments of MM, including bortezomib [5], melphalan [2], cyclophosphamide [2], or thalidomide [6]. The patient had been started on lenalidomide 1 week prior to receiving elotuzumab. However, no significant changes in his labs occurred after starting lenalidomide, and he had tolerated it 1 month prior with respect to his renal function and electrolytes. Additionally, the patient was likely volume depleted and had recently ingested NSAIDs. However, we find these factors to be unlikely to cause such a profound level of hyperuricemia. Moreover, the patient did not respond to volume resuscitation, making hypovolemia a less likely cause of AKI. While these factors may have contributed to the propagation of our patient’s acute renal failure, we find it unlikely that they were the initial instigating factors. Given the presence of uric acid crystals on urine microscopy, the most likely explanation for the patient’s kidney injury was his profound hyperuricemia leading to a urate crystal nephropathy. While many of the patients in the studies listed [2, 5, 6] had elevated uric acid levels, the uric acid level from our patient was excessively elevated, and uric acid crystals were noted on his urine microscopy as well as non-obstructing stones on his renal ultrasound. To our knowledge, elotuzumab does not have any direct effect on uric acid metabolism. 

The patient was also noted to have significantly elevated prothrombin time (PT), partial thromboplastin time (PTT), with low platelets and was eventually complicated by ongoing epistaxis. These parameters remained low in spite of ongoing supplementation. This was likely related to the patient’s supratherapeutic dabigatran levels. Dabigatran is renally cleared and likely accumulated in our patient secondary to his AKI. Dabigatran has been shown to be cleared by high-flux intermittent hemodialysis with ~ 50 – 70% reduction in plasma levels [9]. However, significant rebound in plasma dabigatran levels usually occurs after intermittent hemodialysis, likely secondary to dabigatran’s large volume of distribution [9]. CVVHDF has also been shown to significantly reduce dabigatran levels, up to 80% over a 30-hour period [9]. Similar results were shown in a second case, in which dabigatran levels were decreased by 85% over a 12-hour period [10]. Since we could expect reasonable clearance of dabigatran with CVVHDF, and given that our patient was hypotensive on presentation, we decided to proceed with CVVHDF as opposed to intermittent hemodialysis. Given that our patient’s clinical condition continued to decline resulting in eventual withdrawal of care, repeat dabigatran levels were not obtained. 

As above, this likely represents the first published case of TLS with elotuzumab, which was likely secondary to the underlying hyperproliferative state of the patient’s underlying MM. An additional teaching point from this case is that, while TLS is a relatively rare complication of MM, clinicians should be aware of their patient’s underlying disease burden and/or proliferative state – as these are the most likely risk factors for the development of TLS in MM –, which could potentially allow for the timely diagnosis and/or institution of preventative measures in high-risk patients. 

## Funding 

Dr. Atchinson is currently receiving funding from the University of Michigan, Department of Nephrology, NIDDK training grant, T32DK007378. 

## Conflict of interest 

The authors have no conflicts of interest to declare. 

**Table 1.** Serum labs pre- and post-elotuzumab infusion.

Serum parameter (units)	Pre-elotuzumab (November 15, 2016)	Post-elotuzumab (November 22, 2016)
Sodium (mmol/L)	137	133
Potassium (mmol/L)	4.5	6.2
Chloride (mmol/L)	102	93
CO_2_ (mmol/L)	27	23
Urea nitrogen (mg/dL)	18	93
Creatinine (mg/dL)	1.26	9.51
Glucose (mg/dL)	134	104
Calcium (mg/dL)	9.9	9.7
Protein (g/L)	8.5	8.8
Albumin (g/L)	3.2	3.2
AST (IU/L)	71	68
ALT (IU/L)	15	43
Alkaline phosphatase (IU/L)	123	326
Total bilirubin (mg/dL)	0.7	1.4
Phosphorus (mg/dL)	Not checked	4.9
Uric acid (mg/dL)	Not checked	35.9
Lactate dehydrogenase (IU/L)	Not checked	606
Lactate (mmol/L)	Not checked	0.8
White blood cells (K/µL)	5.8	4.0
Hemoglobin (g/dL)	9.9	7.7
Platelets (K/µL)	83	30
Plasmacytes (% of CBC differential)	4.0	7.0
Haptoglobin (mg/dL)	Not checked	251
INR	Not checked	5.7
PTT (sec)	Not checked	58
Fibrinogen (mg/dL)	Not checked	345
Dabigatran (µg/mL)	Not checked	> 1.0

AST = aspartate aminotransferase; ALT = alanine aminotransferase; PTT = partial thromboplastin time.

## References

[1] Korbet SM Schwartz MM Multiple myeloma. J Am Soc Nephrol. 2006; 17: 2533–2545. 16885408 10.1681/ASN.2006020139

[2] Fassas AB Desikan KR Siegel D Golper TA Munshi NC Barlogie B Tricot G Tumor lysis syndrome complicating high-dose treatment in patient with multiple myeloma. 1999; 105: 938–941. 10.1046/j.1365-2141.1999.01467.x 10554803

[3] Cairo MS Bishop M Tumour lysis syndrome: new therapeutic strategies and classification. Br J Haematol. 2004; 127: 3–11. 15384972 10.1111/j.1365-2141.2004.05094.x

[4] Oiwa K Morita M Kishi S Okura M Tasaki T Matsuda Y Tai K Hosono N Ueda T Yamauchi T High risk of tumor lysis syndrome in symptomatic patients with multiple myeloma with renal dysfunction treated with bortezomib. Anticancer Res. 2016; 36: 6655–6662. 27919998 10.21873/anticanres.11274

[5] Wang L Jian Y Yang G Gao W Wu Y Zuo L Management of tumor lysis syndrome in patients with multiple myeloma during bortezomib treatment. Clin J Oncol Nurs. 2015; 19: E4–E7. 25689664 10.1188/15.CJON.E4-E7

[6] Fuente N Mañe JM Barcelo R Muñoz A Perez-Hoyos T Lopez-Vivanco G Tumor lysis syndrome in a multiple myeloma treated with thalidomide. Ann Oncol. 2004; 15: 537. 10.1093/annonc/mdh116 14998862

[7] Lonial S Dimopoulos M Palumbo A White D Grosicki S Spicka I Walter-Croneck A Moreau P Mateos MV Magen H Belch A Reece D Beksac M Spencer A Oakervee H Orlowski RZ Taniwaki M Röllig C Einsele H Wu KL Elotuzumab therapy for relapsed or refractory multiple myeloma. N Engl J Med. 2015; 373: 621–631. 26035255 10.1056/NEJMoa1505654

[8] Berdeja J Jagannath S Zonder J Badros A Kaufman JL Manges R Gupta M Tendolkar A Lynch M Bleickardt E Paliwal P Vij R Pharmacokinetics and safety of elotuzumab combined with lenalidomide and dexamethasone in patients with multiple myeloma and various levels of renal impairment: results of a phase Ib study. Clin Lymphoma Myeloma Leuk. 2016; 16: 129–138. 26795075 10.1016/j.clml.2015.12.007 PMC6857171

[9] Singh T Maw TT Henry BL Pastor-Soler NM Unruh ML Hallows KR Nolin TD Extracorporeal therapy for dabigatran removal in the treatment of acute bleeding: a single center experience. Clin J Am Soc Nephrol. 2013; 8: 1533–1539. 23704302 10.2215/CJN.01570213 PMC3805068

[10] Parli SE Thompson Bastin ML Lewis DA Use of continuous renal replacement therapy for removal of dabigatran in a patient in need of emergent surgery. Case Rep Crit Care. 2016; 2016: 9692568. 27313909 10.1155/2016/9692568 PMC4899578

